# Bioactive adrenomedullin in sepsis patients in the emergency department is associated with mortality, organ failure and admission to intensive care

**DOI:** 10.1371/journal.pone.0267497

**Published:** 2022-04-28

**Authors:** Oscar H. M. Lundberg, Mari Rosenqvist, Kevin Bronton, Janin Schulte, Hans Friberg, Olle Melander

**Affiliations:** 1 Department of Clinical Sciences, Anaesthesiology and Intensive Care, Medical Faculty, Lund University, Lund, Sweden; 2 Department of Intensive and Perioperative Care, Skåne University Hospital, Malmö, Sweden; 3 Department of Clinical Sciences, Medical Faculty, Lund University, Malmö, Sweden; 4 Department of Infectious Diseases, Skåne University Hospital, Malmö, Sweden; 5 Department of Internal Medicine, Skåne University Hospital, Malmö, Sweden; 6 SphingoTec GmbH, Hennigsdorf, Germany; Taipei Medical University School of Medicine, TAIWAN

## Abstract

**Background:**

Adrenomedullin is a vasoactive hormone with potentially prognostic and therapeutic value, which mainly has been investigated in intensive care unit (ICU) settings. The triaging in the emergency department (ED) of patients to the right level of care is crucial for patient outcome.

**Objectives:**

The primary aim of this study was to investigate the association of bioactive adrenomedullin (bio-ADM) with mortality among sepsis patients in the ED. Secondary aims were to investigate the association of bio-ADM with multiple organ failure (MOF), ICU admission and ED discharge.

**Methods:**

In this prospective observational cohort study, adult sepsis patients in the ED (2013–2015) had blood samples collected for later batch analysis of bio-ADM. Odds ratios (OR) with 95% confidence interval (CI) for bio-ADM were calculated.

**Results:**

Bio-ADM in 594 sepsis patients was analyzed of whom 51 died within 28 days (8.6%), 34 developed severe MOF, 27 were ICU admitted and 67 were discharged from the ED. The median (interquartile range) bio-ADM was 36 (26–56) and 63 (42–132) pg/mL among survivors and non-survivors, respectively, 81 (56–156) pg/mL for patients with severe MOF and 77 (42–133) pg/mL for ICU admitted patients. Each log-2 increment of bio-ADM conferred an OR of 2.30 (95% CI 1.74–3.04) for mortality, the adjusted OR was 2.39 (95% CI 1.69–3.39). The area under the receiver operating characteristic curve of a prognostic mortality model based on demographics and biomarkers increased from 0.80 to 0.86 (p = 0.02) when bio-ADM was added. Increasing bio-ADM was associated with severe MOF, ICU admission and ED discharge with adjusted ORs of 3.30 (95% CI 2.13–5.11), 1.75 (95% CI 1.11–2.77) and 0.46 (95% CI 0.32–0.68), respectively.

**Conclusion:**

Bio-ADM in sepsis patients in the ED is associated with mortality, severe MOF, ICU admission and ED discharge, and may be of clinical importance for triage of sepsis patients in the ED.

## Introduction

### Background

Sepsis is a life-threatening condition which comes in a variety of shapes and severities, affecting millions of people worldwide [1]. In spite of improvements in recent years, the mortality of the most severe form of sepsis, septic shock, is still unacceptably high, up to 38% in North America and Europe [2].

The success rate of treating sepsis is time-sensitive, a short time to recognition and treatment is fundamental for outcomes, exemplified by the recommendation to consider one-hour bundles [3].

Identification of patients with sepsis in the emergency department (ED) is difficult and triaging patients to the correct level of care is a challenge. Biomarkers may be of help in identifying and stratifying sepsis according to severity of disease. An optimal biomarker in the ED setting should thus offer a method to distinguish individuals who can return home from those at high risk of developing multiple organ failure (MOF), thereby guiding clinicians to ensure patients an adequate level of care.

### Adrenomedullin

Adrenomedullin (ADM) is a hormone produced by a variety of different cell types and was first derived from pheochromocytoma nearly three decades ago [4]. ADM has homeostatic and regulating effects on renal, immunological, endocrine and cardiovascular systems [4–7]. The effects of ADM on blood vessels include vasodilation [8] and stabilization of the barrier function of endothelial cells maintaining adequate permeability [9, 10]. ADM is typically elevated in patients with the metabolic syndrome [11], heart failure [12–15], chronic kidney failure [16–18] as well as in unselected critically ill patients [19, 20].

There are two predominant methods to measure ADM in peripheral blood. One is based on a part of the pre-cursor pro-hormone of ADM–mid regional pro-adrenomedullin (MR-proADM) [21], while the other measures bioactive adrenomedullin (bio-ADM) directly [22]. Few studies have described the correlation between measured MR-proADM and bio-ADM [22–24]. Although MR-proADM shows prognostic value in disease, it has no known action by itself, which makes the measurement of bio-ADM more attractive and clinically relevant. A median bio-ADM concentration of 20.7 pg/mL with 43 pg/mL as the 99^th^ percentile among 200 healthy subjects has been reported [22].

### Adrenomedullin in sepsis

Several studies have reported a strong association between elevated ADM levels and mortality, severity of illness and need for organ support in sepsis patients, using either of the two methods [19, 22, 25–31], proposing ADM to be a predictive biomarker in sepsis. Our group has recently reported that bio-ADM may be a specific marker of sepsis in a general intensive care unit (ICU) population [19].

In addition, the potential of modulating the ADM hormonal system has gained interest since exogenous infusion of ADM in animal models of sepsis has been shown to improve outcomes [10, 32, 33], which has led to the hypothesis that an increment of intravascular bio-ADM may be of therapeutic value in sepsis [9]. This has led to studies of the non-neutralizing anti-ADM antibody Adrecizumab in humans [23, 24, 34]. The formation of Adrecizumab-ADM complexes generates elevated intravascular bio-ADM concentrations where ADM can exert its endothelium-stabilizing effects [9, 35]. The increase of bio-ADM, on the other hand, is not accompanied by an elevation of MR-proADM suggesting a redistribution of ADM rather than an increased synthesis [23, 24]. The clinical implication of the use of Adrecizumab in sepsis is yet unanswered, but clinical trials to investigate this are planned [36].

While most of the studies describing ADM in sepsis are derived from ICU settings, similar findings have been found in populations originating in the ED. Studies performed on infected patients in the ED have reported MR-proADM to have a higher association with mortality and ICU admission compared to other commonly used biomarkers and clinical scores [37–39]. Further, a combination of MR-proADM with clinical scores and other biomarkers in order to improve prognostic accuracy has also been proposed [40–43].

Studies measuring bio-ADM in the ED are sparse. Two recent studies have described bio-ADM in ED populations but patients presented with either acute heart failure or dyspnea [14, 44]. The original paper presenting bio-ADM [22], however, analyzed bio-ADM in patients with suspected sepsis in the ED. In the present study, our aim was to investigate the prognostic capability of bio-ADM in a large sepsis cohort in the ED.

### Objectives

We hypothesized that increasing levels of bio-ADM in sepsis patients in the ED were associated with subsequent severity of sepsis and increased mortality.

The primary aim of this study was to investigate the association of bio-ADM with 28-day mortality. Secondary aims were to *I*) assess whether bio-ADM could improve the prognostic precision of a mortality prediction model, *II*) compare the prognostic properties of bio-ADM with other commonly used biomarkers, and III) investigate the association of bio-ADM with *a)* severe MOF, *b*) ICU admission among patients with no limitations of care and *c*) ED discharge.

## Material and methods

### Study design and setting

This single center prospective observational cohort study was performed in the ED of Skåne University Hospital in Malmö, Sweden. With a catchment population of 400000, the hospital has approximately 85000 emergency visits per year.

Both oral and written consent was obtained by the patients or by their next of kin after they had the opportunity to read and review a written description of the study design and purpose. If a patient at inclusion had a decreased level of consciousness, consent was obtained retrospectively. This consent procedure and the study as a whole, was approved by the Regional Ethical board in Lund (DNR 2013/635).

The STROBE guidelines were followed [45].

### Participants

Between December 2013 and February 2015, patients 18 years or older, seeking care during office hours (Monday to Friday, 6 AM to 6 PM) in the ED, were screened for inclusion by trained research nurses. The inclusion criteria were based on the sepsis definition at the time [46]: suspected infection in addition to two or more systemic inflammatory response syndrome (SIRS) criteria. Inclusion criteria were: 1) a body temperature lower than 36°C, or higher than 38°C, or self-reported fever/chills within 24 hours preceding the ED visit, 2) a respiratory rate higher than 20 breaths/min, 3) a heart rate higher than 90 beats/min. White blood cell count was not part of the inclusion criteria due to unavailability at the time of screening.

The study size was not predefined and consisted of a convenience sample of patients included during the study period.

### Variables

The primary outcome was 28-day mortality. Secondary outcomes were number of failing organ systems, ICU admission and ED discharge. Failing organ systems, defined in S1 Table, were registered up to 48 hours after presentation at the ED and trichotomized into 1) no organ failure, 2) intermediate organ failure (one to three failing organ systems) and 3) severe MOF (four or more failing organ systems). ICU admission was registered during the entire follow-up time. Furthermore, a prognostic baseline model including covariates with significantly different distribution in relation to 28-day mortality, and three commonly used biomarkers, lactate, C-reactive protein (CRP) and creatinine was created to investigate whether the addition of bio-ADM improved the model. Premorbid comorbidities were registered and classified as shown in S2 Table.

### Data sources

Patient demographics and comorbidities were systematically and prospectively collected from medical records which were reviewed by infectious disease physicians. Site of infection and type of ward, if admitted to the hospital, were recorded.

### Biomarkers

Blood was drawn peripherally within one hour of presentation to the ED. All biomarkers except for bio-ADM were analyzed routinely in the certified hospital laboratory. For the analysis of bio-ADM plasma ethylenediaminetetraacetic acid plasma samples were frozen within 2 hours and stored at -80˚C until later batch analysis. Measurements of bio-ADM was undertaken at the laboratory of SphingoTec GmbH in Hennigsdorf, Germany in June 2018 as described elsewhere [47].

### Statistics

For all hypotheses tests, we considered p-values <0.05 as significant. Group comparisons of continuous variables were performed using Wilcoxon rank-sum test (Mann-Whitney U test) for two groups. If there were more than two groups to be compared, Kruskal-Wallis rank sum test was used, and if significant, a comparison with pairwise Wilcoxon test, with Holm´s procedure for adjustment for multiple testing was performed. Differences in proportions were assessed using Pearson’s X^2^ test. Medians were reported with their corresponding interquartile ranges (IQR). Uni- and multivariable binary logistic regression was used to analyze outcomes. Covariates in the multivariable binary logistic regression analyses were included if they were significantly differently distributed in relation to the primary outcome. The results of the regression analyses were reported as odds ratios (OR) with 95% confidence intervals (CI). The regression models were evaluated with the Hosmer-Lemeshow goodness-of-fit test with ten groups, and only models resulting in non-significant tests were reported [48]. Body mass index (BMI) was stratified according to underweight (<18.5), normal (18.5–25), overweight (25–30) and obese (>35) prior to inclusion in the multivariable binary logistic regressions, with the normal group as reference. If a parameter, due to skewness, needed transformation, the base 2 logarithm was used. The difference in Kaplan-Meier curves was evaluated with the log-rank test [49]. Areas under the receiver operating characteristic curve (AUROC) were calculated [50]. Differences in AUROCs were tested with the method of DeLong et al [51]. Admissions with missing data were excluded from calculations. If a variable had missing values (MV) these were specified. R Studio version 1.2.1335 was used as statistical software.

## Results

### Participants

**Fig 1:**
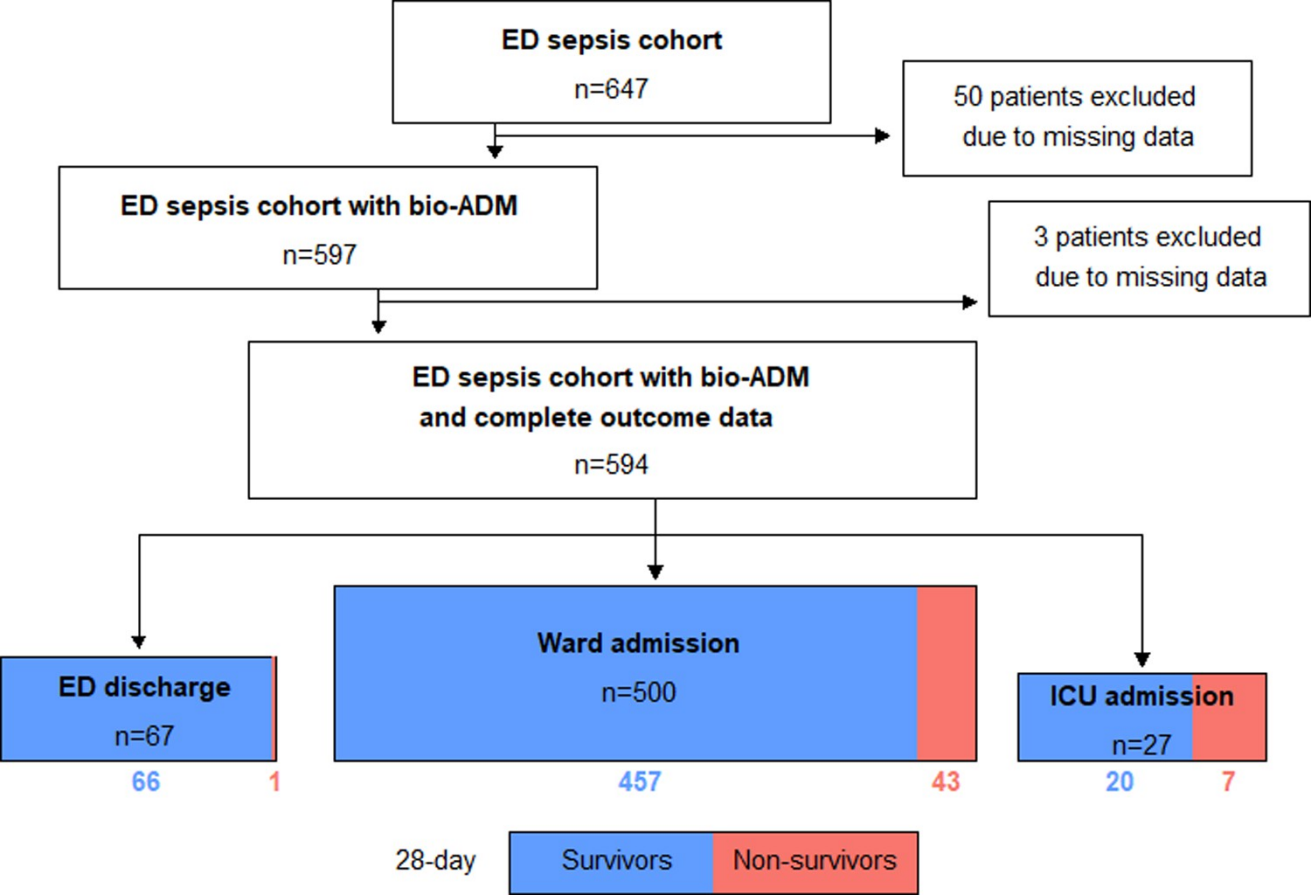
Patient flowchart according to inclusion eligibility, referral after assessment in the emergency department and 28-day mortality. In total 53 patients were excluded due to missing plasma and missing outcome data as 28-day mortality, organ failure and ICU admission. *ED*: *emergency department; bio-ADM*: *bioactive adrenomedullin; ICU*: *intensive care unit*.

Inclusion criteria were met by 647 patients. Due to missing data 50 patients were excluded and bio-ADM was analyzed in 597 patients. Of these, three additional patients had missing mortality follow up data leaving 594 subjects to be included in the study, see Fig 1.

### Demographics

Demographics including age, sex, comorbidities and site of infection are shown in Table 1. Non-survivors were generally older, had a lower BMI and a higher burden of cardiovascular disease. Further, non-survivors more often had a decision on limitation of care. The site of infection varied, non-survivors had a higher rate of pulmonary infections, whereas infections refrained to the upper respiratory tract and urinary sites were more common among survivors.

**Table 1:** Demographics and outcomes of the sepsis cohort and comparisons between 28-day non-survivors and survivors.

Baseline characteristics	Sepsis cohort	Non-survivors	Survivors	p-value
Number, n (% of Sepsis cohort)	594 (100)	51 (8.6)	543 (91.4)	
Age in years, median (IQR)	73 (61–82)	80 (73–88)	72 (59–82)	<0.001
Female sex, n (%)	289 (48.6)	22 (43.1)	267 (49.2)	0.50
Body mass index (MV = 27), median (IQR)	25.7 (22.5–29.6)	24.0 (21.7–27.9)	25.8 (22.6–30)	0.05
**Comorbidities**				
Cardiovascular disease (MV = 2), n (%)	229 (38.7)	316 (60.8)	198 (36.6)	0.001
Respiratory disease (MV = 2), n (%)	140 (23.6)	18 (35.3)	122 (22.6)	0.06
Neurological disease (MV = 1), n (%)	98 (16.5)	9 (17.6)	89 (16.4)	0.98
Renal disease, n (%)	45 (7.6)	5 (9.8)	40 (7.4)	0.72
Cancer (n = 591), n (%)	165 (27.9)	20 (40)	145 (26.8)	0.13
Immunodeficiency (MV = 9), n (%)	32 (5.5)	6 (12)	26 (4.9)	0.07
Diabetes (MV = 1), n (%)	114 (19.2)	15 (29.4)	99 (18.2)	0.08
Psychiatric disorder (MV = 2), n (%)	63 (10.6)	4 (8)	59 (10.9)	0.69
None of those listed above, n (%)	146 (24.6)	4 (7.8)	142 (26.1)	0.006
Limitation of care (MV = 5), n (%)	90 (15.3)	24 (47.1)	66 (12.3)	<0.001
**Site of infection**				
Pulmonary, n (%)	199 (33.5)	24 (47.1)	175 (32.2)	0.02
URTI, n (%)	52 (8.8)	0 (0)	52 (9.6)	0.05
Urinary, n (%)	129 (21.7)	4 (7.8)	125 (23)	0.03
Bone and joint, n (%)	7 (1.2)	1 (2.0)	6 (1.1)	1
SSTI, n (%)	58 (9.8)	7 (13.7)	51 (9.9)	0.36
Gastrointestinal, n (%)	23 (3.9)	1 (2.0)	22 (4.1)	0.78
Other, n (%)	76 (12.8)	7 (13.7)	69 (12.7)	0.87
No confirmed infection, n (%)	50 (8.4)	7 (13.7)	43 (7.9)	0.24
**Outcomes**				
No organ failure, n (%)	278 (46.8)	8 (15.7)	270 (49.7)	<0.001
Intermediate organ failure (1–3), n (%)	282 (47.5)	30 (58.8)	252 (46.4)	0.12
Severe MOF (≥4), n (%)	34 (5,7)	13 (25.4)	21 (3.9)	<0.001
ICU admission, n (%)	27 (4.5)	7 (13.7)	20 (3.7)	0.003
Discharged from ED, n (%)	67 (11.3)	1 (2.0)	66 (12.2)	0.05
**Biomarkers**				
Bio-ADM pg/mL, median (IQR)	38 (27–60)	63 (42–132)	36 (26–56)	<0.001
Lactate (MV = 25) mmol/L, median (IQR)	1.7 (1.3–2.7)	2.1 (1.3–3.0)	1.7 (1.2–2.6)	0.11
CRP (MV = 7) mg/L, median (IQR)	72 (25–160)	100 (51–178)	69 (23–156)	0.04
Creatinine (MV = 5) μmol/L, median (IQR)	87 (68–120)	105 (79–160)	85 (68–117)	0.006

Data regarding general characteristics, comorbidities, site of infection, outcomes and biomarkers are presented. Non-survivors were compared to survivors, and the p-values refer to that comparison. Proportions (%) are within their subgroups unless otherwise specified. *IQR*: *interquartile range; MV*: *missing values*, *URTI*: *upper respiratory tract infection; SSTI*: *skin and soft tissue infection; ED*: *emergency department; MOF*: *multiple organ failure; ICU*: *intensive care unit; bio-ADM*: *bioactive adrenomedullin; CRP*: *C-reactive protein*

### Outcomes

Fifty-one patients (8.6%) died within 28 days, of whom 25 patients (4.2%) died within 7 days. Among 316 patients who developed organ failure (53.2%), 34 patients (5.7%) developed severe MOF as shown in Table 1. Twenty-seven patients (4.5%) were admitted to the ICU. Just over every tenth patient (11.3%) was discharged directly from the ED. One of them, the only 28-day non-survivor in the group, was offered admission to the ICU but declined and was discharged to palliative care at home after discussion with the patient and the patient´s family.

### Bio-ADM

Levels of bio-ADM ranged 8–813 pg/mL and were logarithmically transformed due to skewness.

#### Bio-ADM and mortality

**Fig 2:**
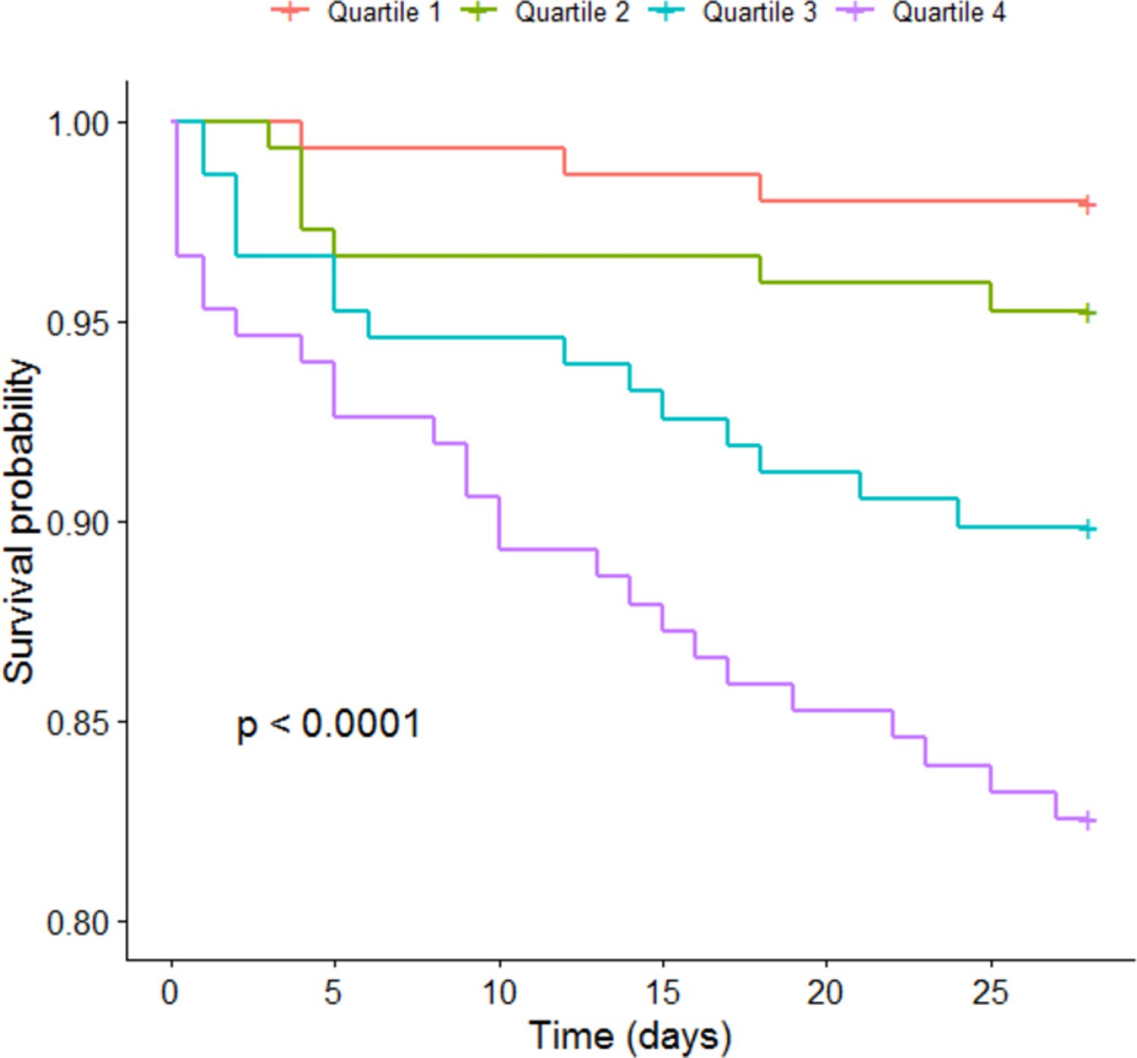
Kaplan-Meier curve according to quartiles of bio-ADM and 28-day mortality. The range of bio-ADM (pg/mL) was for Quartile 1: <27; Quartile 2: 27–38; Quartile 3: 38–60; Quartile 4: >60. The p-value was derived from the log-rank test. *bio-ADM*: *bioactive adrenomedullin*.

Non-survivors had higher levels of bio-ADM than survivors, 63 (42–132) pg/mL versus 36 (26–56) pg/mL, see Table 1. Dividing the patients into quartiles based on levels of bio-ADM a significant separation between the corresponding Kaplan-Meier curves for 28-day mortality, was observed, see Fig 2. The association of bio-ADM with 28-day mortality showed a univariate OR of 2.30 (95% CI 1.74–3.04), which remained significant after adjustments, 2.39 (95% CI 1.69–3.39), see Table 2.

**Table 2:** Odds ratios for bio-ADM from uni- and multivariate binary logistic regression analyses for primary and secondary outcomes.

Univariate				Multivariate			
Primary outcome	OR	95% CI	p-value	Primary outcome	OR	95% CI	p-value
28-day mortality	2.30	1.74–3.04	<0.001	28-day mortality (MV = 29)	2.39	1.69–3.39	<0.001
**Secondary outcome**	**OR**	**95% CI**	**p-value**	**Secondary outcome**	**OR**	**95% CI**	**p-value**
Severe MOF	3.22	2.26–4.59	<0.001	Severe MOF (MV = 29)	3.30	2.13–5.11	<0.001
ICU admission (MV = 5)	2.21	1.50–3.24	<0.001	ICU admission (MV = 27)	1.75	1.11–2.77	0.02
ED discharge	0.41	0.29–0.56	<0.001	ED discharge (MV = 29)	0.46	0.32–0.68	<0.001

The odds ratio for bio-ADM was calculated on a base 2 logarithmic scale. Multivariate included covariates bio-ADM, age, known cardiovascular disease, BMI, urinary, URTI and pulmonary site of infection. The outcome ICU admission was only calculated among patients with no limitations of care (n = 499). *bio-ADM*: *bioactive adrenomedullin; BMI*: *body mass index; ICU*: *intensive care unit; URTI*: *upper respiratory tract infection; OR*: *odds ratio; CI*: *confidence interval; MV*: *missing values; ED*: *emergency department*.

**Fig 3:**
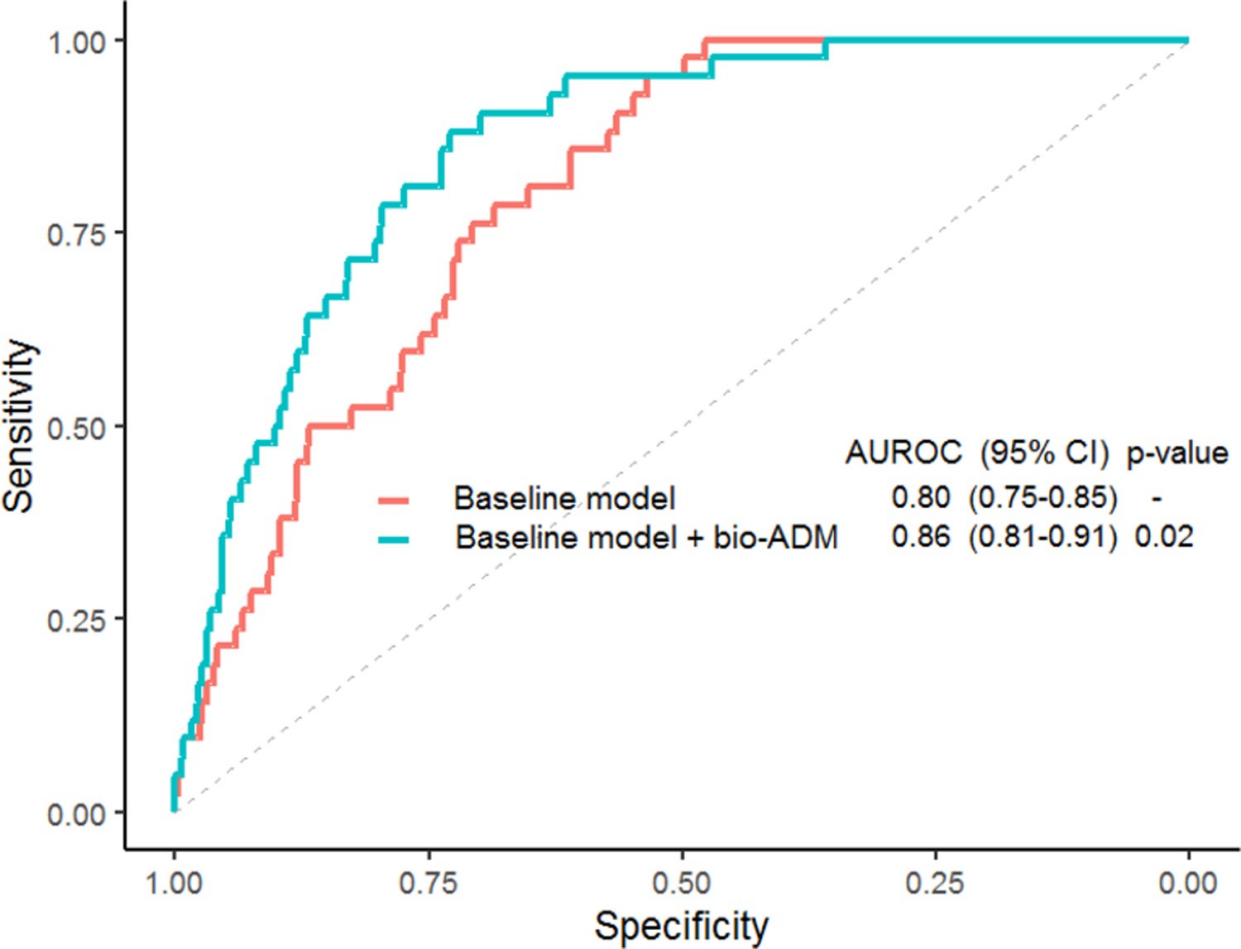
Receiver operating characteristics curves for mortality predictive models. Baseline model with covariates age, known cardiovascular, BMI, URTI, urinary and pulmonary site of infection, C-reactive protein, lactate and creatinine. The additive value of bio-ADM is shown in Baseline + bio-ADM. The p-value is derived from the DeLong’s test for comparison between the two AUROCs. *BMI*: *body mass index; URTI*: *upper respiratory tract infections; bio-ADM*: *bioactive adrenomedullin; AUROC*: *area under the receiver operating characteristic; CI*: *confidence interval*.

A baseline mortality prediction model including age, previous cardiovascular disease, BMI, URTI, urinary or pulmonary infection site and routine biomarkers (CRP, lactate, creatinine) resulted in an AUROC of 0.80, which significantly improved with the addition of bio-ADM to an AUROC of 0.86 (p = 0.02), see Fig 3.

#### Bio-ADM and other biomarkers

**Fig 4:**
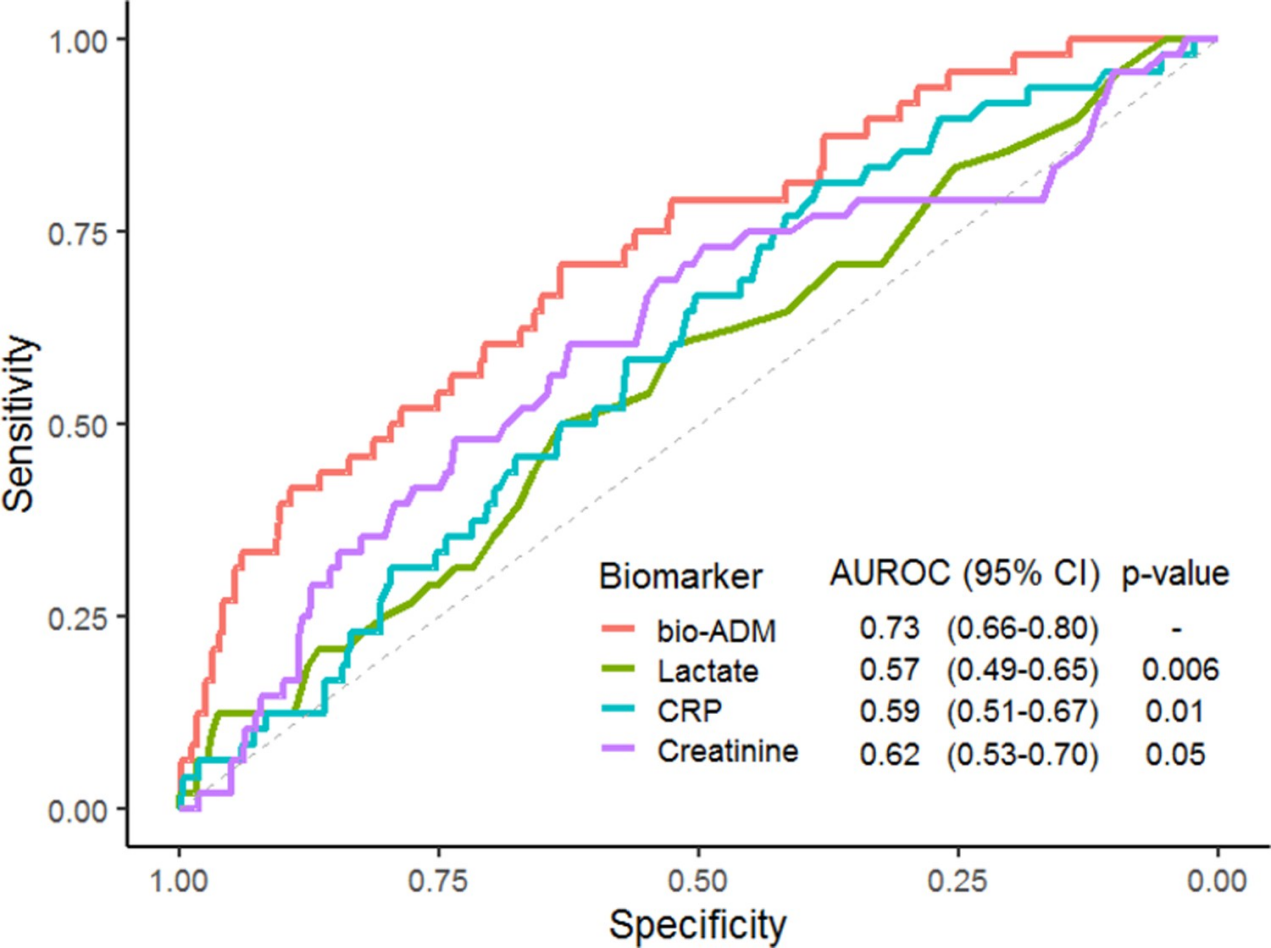
Receiver operating characteristics curves for the biomarkers bio-ADM, lactate, CRP and creatinine corresponding to 28 day mortality. Only patients with all four biomarkers analyzed were included (n = 562). P-values are derived from the DeLong’s test for comparison with the AUROC of bio-ADM. *bio-ADM*: *bioactive adrenomedullin; CRP*: *C-reactive protein; AUROC*: *area under the receiver operating characteristic curve*.

The receiver operating characteristics curves with corresponding AUROCs for lactate, CRP, creatinine and bio-ADM in relation to 28-day mortality are shown in Fig 4. Bio-ADM had a significantly higher AUROC than lactate, CRP and creatinine.

#### Bio-ADM and organ failure

**Fig 5:**
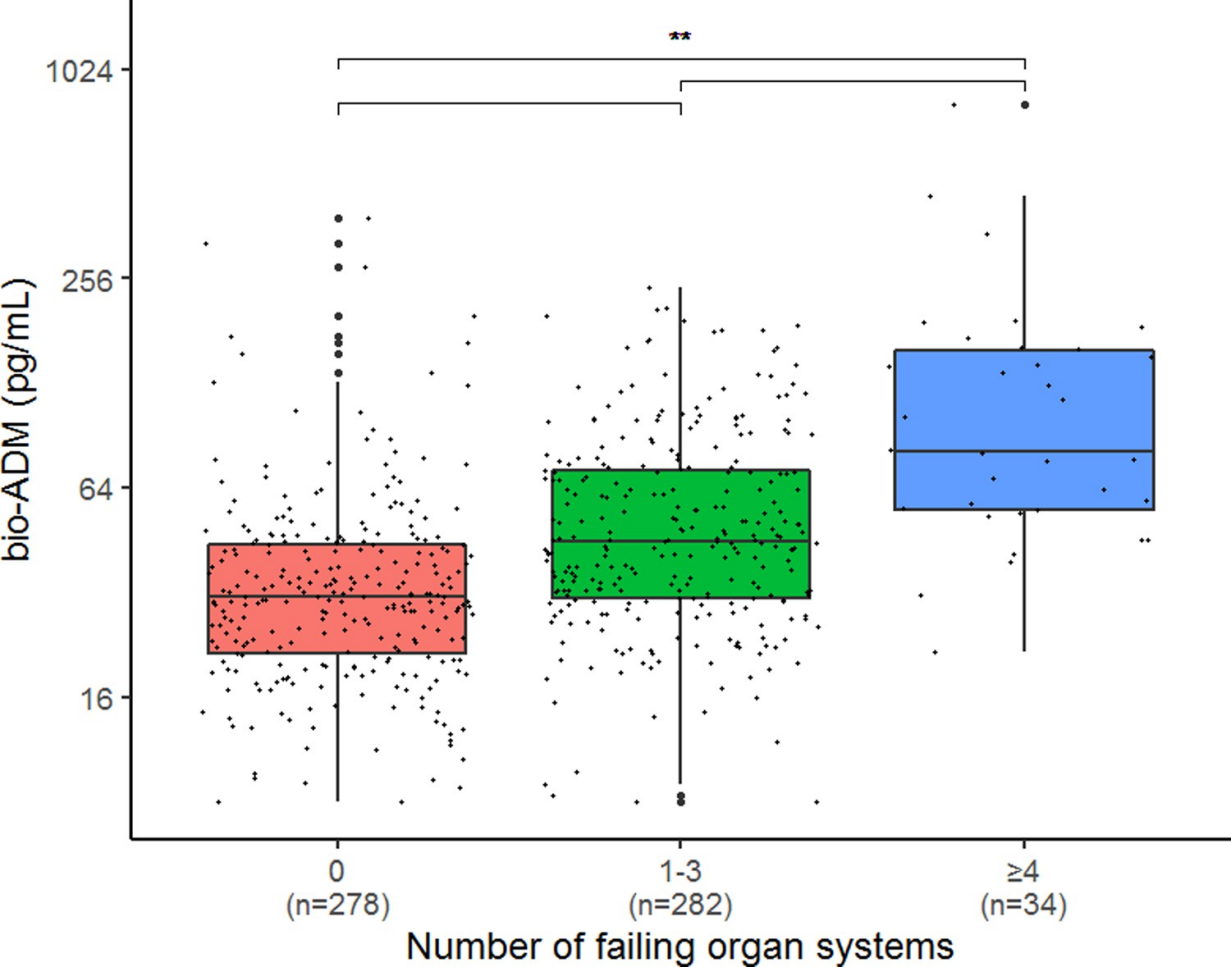
Boxplots showing levels of bio-ADM according to number of failing organ systems. P-values are derived from the pairwise Wilcoxon test. **: p<0.001 *bio-ADM*: *bioactive adrenomedullin*.

Bio-ADM concentrations among patients without organ failure, 31 (21–44) pg/mL, intermediate organ failure, 45 (31–72) pg/mL, and severe MOF, 81 (56–156) pg/mL, are shown in Fig 5. A significant separation between the groups was seen (p<0.001).

ORs from uni- and multivariate regressions for bio-ADM for the development of severe MOF were 3.22 (95% CI 2.26–4.59) and 3.30 (95% CI 2.13–5.11), respectively, see Table 2.

#### Bio-ADM and ICU admission

**Fig 6:**
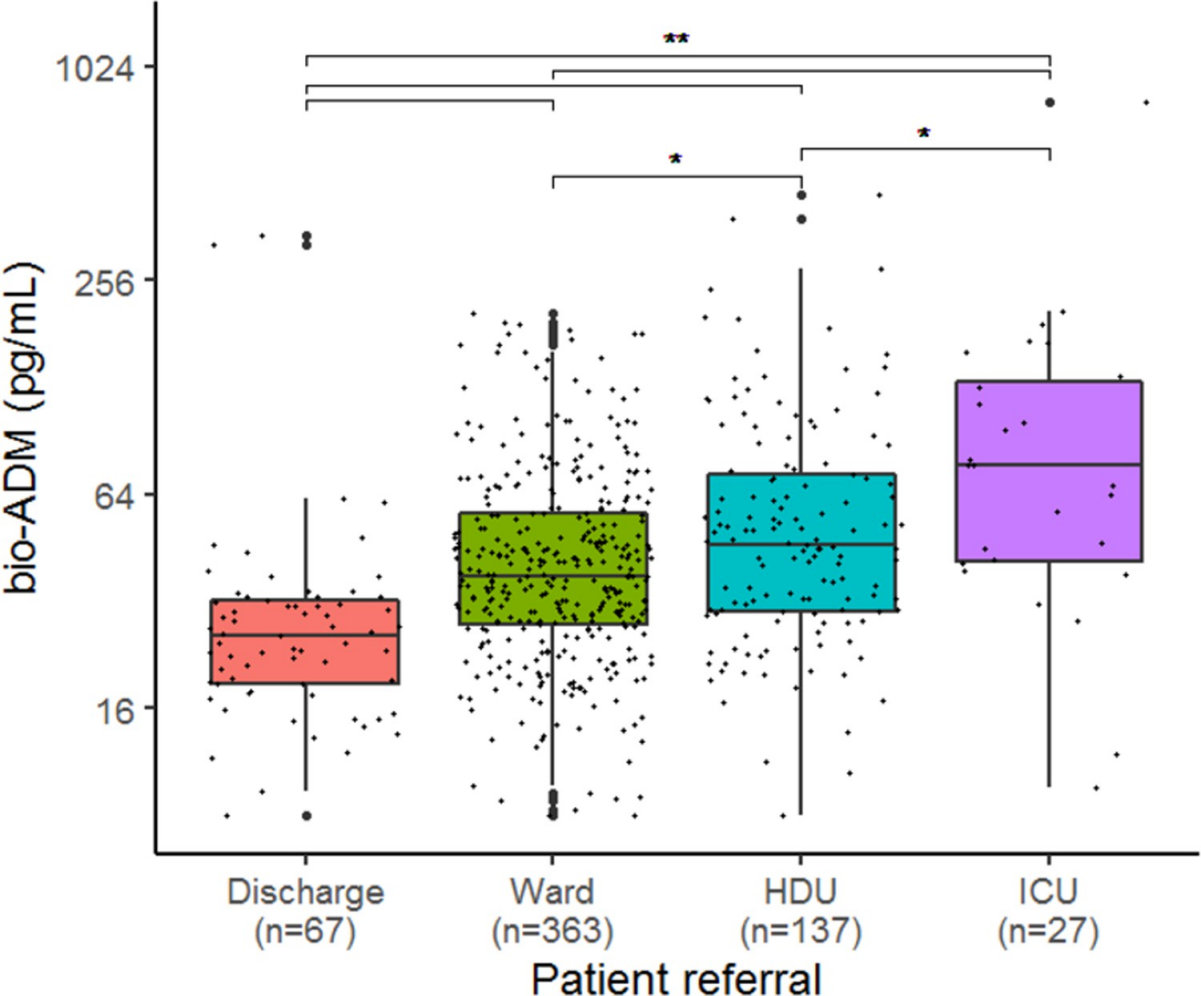
Boxplots showing levels of bio-ADM according to patient referral after assessment in the emergency department. P-values are derived from the pairwise Wilcoxon test. *: p<0.05, **: p<0.001. *bio-ADM*: *bioactive adrenomedullin; HDU*: *high dependency unit; ICU*: *intensive care unit*.

Patients admitted to the ICU had significantly higher levels of bio-ADM, 77 (42–133) pg/mL, than patients not admitted to the ICU, 41 (28–61) pg/mL, and patients discharged from the ED, 26 (19–32) pg/mL (p<0.001). Fig 6 shows the distribution of bio-ADM according to patient referral after assessment in the ED. The distribution was significantly separated between the groups (p<0.001). There was a significant association between ICU admission and increasing levels of bio-ADM, both before and after adjustment, see Table 2.

#### Bio-ADM and ED discharge

The median bio-ADM among patients discharged from ED was 26 (19–32) pg/mL, significantly lower than the corresponding median of 41 (28–63) pg/mL among patients admitted to a hospital ward or admitted to the ICU, 73 (41–130) pg/mL (p<0.001).

Uni- and multivariate logistic regression analyses showed an inverse association of increasing levels of bio-ADM and ED discharge, see Table 2.

## Discussion

To our knowledge, this is the largest study to date investigating bio-ADM as a prognostic biomarker in patients with sepsis in the ED. Our data show that high levels of bio-ADM in the ED are associated with mortality, development of severe MOF and referral to intensive care. Moreover, we found that bio-ADM adds important prognostic information to the commonly used prognostic factors age, comorbidities, site of infection and routine biomarkers, and that low levels of bio-ADM are related to less severe disease and discharge from the ED.

Our study suggests that bio-ADM is of potential clinical use for early stratification of unselected sepsis patients in the ED. Alongside with the first study describing bio-ADM [22] and recent reports on possible applications of bio-ADM in patients with dyspnea [44] as well as heart failure [14], our data show that bio-ADM is a potentially important clinical biomarker in the ED. Whether these results are generalizable to a broader unselected ED population remains unknown and needs to be addressed in future studies. However, reports where MR-proADM was measured in broader ED populations show promising results [42, 52].

We found a strong association between bio-ADM in the ED and mortality, which remained after adjustments for known prognostic factors. Similar findings have been described in previous studies for both septic [19, 29–31] and non-septic [19, 20] patients treated in the ICU, but not as clearly among septic patients in the ED [22]. The prognostic ability of bio-ADM to predict mortality by itself was modest in the present study, but superior to three commonly used biomarkers, lactate, CRP and creatinine. Importantly, a baseline prediction model was improved when bio-ADM was added, indicating strong additional prognostic properties for bio-ADM. Our findings resemble results from a study in a similar setting where ADM was analyzed using the MR-proADM method. In that study, Scheutz et al. reported an improvement of a predictive model with an increased AUROC from 0.79 to 0.84, when MR-proADM was added [52].

The highest levels of bio-ADM in our study were found among patients admitted who developed severe MOF. Rising levels of bio-ADM were associated with increasing number of failing organ systems in sepsis patients. These results are in line with previous findings that septic patients with high levels of bio-ADM in the ICU had an increased need of organ support [19, 20, 29–31].

Interestingly, in the present ED cohort the median bio-ADM of 73 (41–130) pg/mL in the group of patients admitted to the ICU was similar to the distribution of bio-ADM in an ICU sepsis population where the median bio-ADM was 74 (42–145) pg/mL [19]. This is the first report to describe that bio-ADM is predictive of ICU admission in a sepsis cohort in the ED, which is a novel finding. Due to known variations in the availability of ICU beds across countries, this may however not be generalizable to other hospital environments [53].

The patients discharged from the ED in our cohort had low levels of bio-ADM with levels close to those in healthy subjects [22]. There were some extreme outliers within the group, making a clear threshold of bio-ADM difficult to identify. To our knowledge, no previous study has reported levels of bio-ADM in patients with sepsis discharged from ED.

### Strengths and limitations

This large prospective observational cohort study affirms previous findings from ICU settings and demonstrates the potential applicability of bio-ADM in the ED setting. Furthermore, all patient records in this study were thoroughly revised by infectious disease physicians to assure correct diagnoses. Also, this study included patients with limitations of care.

This study has several limitations. First, we only enrolled participants during office hours which may have led to a selection bias. Second, we were confined to admission samples, making it impossible to analyze dynamic changes and how these could correlate with outcomes. Third, this was a single-center study why generalizability of our results to other hospital settings may be limited. Finally, the study was initiated when sepsis was defined by the Sepsis-2 criteria and thus SOFA score was not recorded.

## Conclusions

Bio-ADM in sepsis patients in the ED is associated with mortality, MOF, ICU admission and ED discharge. Bio-ADM exceeds the prognostic properties of routine biomarkers as lactate, CRP and creatinine and may be of clinical importance for triage of sepsis patients in the ED.

## Supporting information

S1 Table Dysfunction criteria for organ failure up to 48 hours after ED presentation. (DOCX)

S2 Table. Comorbidities and examples of corresponding diagnoses (DOCX)
