# Supplementary material for: Bioactive adrenomedullin in sepsis patients in the emergency department is associated with mortality, organ failure and admission to intensive care
Source: PLoS One. 2022 Apr 28;17(4):e0267497. doi: 10.1371/journal.pone.0267497 (PMC9049572; doi:10.1371/journal.pone.0267497)
Supplement: S1 Table — (DOCX) [file pone.0267497.s001.docx]

**S1 Table. Dysfunction criteria for organ failure up to 48 hours after ED presentation.**

| **Failing organ system** | **Dysfunction criteria** |
| --- | --- |
| Central nervous system | Confusion, drowsiness or loss of consciousness |
| Circulatory failure | Systolic blood pressure < 90 mmHg, mean arterial pressure < 70 mmHg, decrease of systolic blood pressure greater than 40 mmHg or need for vasopressor to maintain blood pressure |
| Respiratory failure | SaO_2_ < 90% or need for mechanical ventilation |
| Kidney failure | Serum creatinine increase of > 44 μmol/L between any two measurements, need for acute renal replacement therapy or an increase in creatinine corresponding to 1.5-fold of baseline with an initial value of > 160 μmol/L within 48 h |
| Liver failure | Total serum bilirubin > 40 μmol/L |
| Hematologic dysfunction | Platelet count < 100 × 10⁹/L, INR > 1.5 or an aPTT > 60 s |
| Metabolic dysfunction | Serum lactate > 3.5 mmol/L. |
