# Supplementary material for: Bioactive adrenomedullin in sepsis patients in the emergency department is associated with mortality, organ failure and admission to intensive care
Source: PLoS One. 2022 Apr 28;17(4):e0267497. doi: 10.1371/journal.pone.0267497 (PMC9049572; doi:10.1371/journal.pone.0267497)
Supplement: S2 Table. Comorbidities and examples of corresponding diagnoses — (DOCX) [file pone.0267497.s002.docx]

**S2 Table. Comorbidities and examples of corresponding diagnoses.**

| **Comorbidities** | **Diseases** |
| --- | --- |
| Cardivascular disease | Ischemic heart disease, heart failure, atrial fibrillation/flutter |
| Respiratory disease | Chronic obstructive pulmonary disease, asthma, restrictive pulmonary disease (fibrosis, interstitial lung disease, asbestosis), other pulmonary disease (including pulmonary hypertension). |
| Neurological disease | Neuromuscular disease (including post-polio syndrome), cerebral stroke, transient ischemic attack, |
| Renal disease | Parenchymatic renal disease, glomerular filtration rate <30 ml/min |
| Psychiatric disorder | Dementia, anxiety, depression, |
